# Dietary Flavones as Dual Inhibitors of DNA Methyltransferases and Histone Methyltransferases

**DOI:** 10.1371/journal.pone.0162956

**Published:** 2016-09-22

**Authors:** Rajnee Kanwal, Manish Datt, Xiaoqi Liu, Sanjay Gupta

**Affiliations:** 1 Department of Urology, Louis Stokes Cleveland Veterans Affairs Medical Center, Cleveland, Ohio, United States of America; 2 Department of Urology, Case Western Reserve University, Cleveland, Ohio, United States of America; 3 The Urology Institute, University Hospitals Case Medical Center, Cleveland, Ohio, United States of America; 4 Center for Proteomics and Bioinformatics, Case Western Reserve University, Cleveland, Ohio, United States of America; 5 Institute of Life Sciences, Ahmedabad University, Gujarat, India; 6 Department of Biochemistry, Purdue University, West Lafayette, Indiana, United States of America; 7 Department of Nutrition, Case Western Reserve University, Cleveland, Ohio, United States of America; 8 Division of General Medical Sciences, Case Comprehensive Cancer Center, Cleveland, Ohio, United States of America; University of California San Francisco, UNITED STATES

## Abstract

Methylation of DNA and histone proteins are mutually involved in the epigenetic regulation of gene expression mediated by DNA methyltransferases (DNMTs) and histone methyltransferases (HMTs). DNMTs methylate cytosine residues within gene promoters, whereas HMTs catalyze the transfer of methyl groups to lysine and arginine residues of histone proteins, thus causing chromatin condensation and transcriptional repression, which play an important role in the pathogenesis of cancer. The potential reversibility of epigenetic alterations has encouraged the development of dual pharmacologic inhibitors of DNA and histone methylation as anticancer therapeutics. Dietary flavones can affect epigenetic modifications that accumulate over time and have shown anticancer properties, which are undefined. Through DNA binding and *in silico* protein-ligand docking studies with plant flavones *viz*. Apigenin, Chrysin and Luteolin, the effect of flavones on DNA and histone methylation was assessed. Spectroscopic analysis of flavones with calf-thymus DNA revealed intercalation as the dominant binding mode, with specific binding to a GC-rich sequence in the DNA duplex. A virtual screening approach using a model of the catalytic site of DNMT and EZH2 demonstrated that plant flavones are tethered at both ends inside the catalytic pocket of DNMT and EZH2 by means of hydrogen bonding. Epigenetic studies performed with flavones exhibited a decrease in DNMT enzyme activity and a reversal of the hypermethylation of cytosine bases in the DNA and prevented cytosine methylation in the GC-rich promoter sequence incubated with the M.SssI enzyme. Furthermore, a marked decrease in HMT activity and a decrease in EZH2 protein expression and trimethylation of H3K27 were noted in histones isolated from cancer cells treated with plant flavones. Our results suggest that dietary flavones can alter DNMT and HMT activities and the methylation of DNA and histone proteins that regulate epigenetic modifications, thus providing a significant anticancer effect by altering epigenetic processes involved in the development of cancer.

## Introduction

The epigenetic activation or inactivation of genes plays a critical role in several human diseases including cancer [1–4]. A major mechanism for gene inactivation is the methylation of CpG islands in genomic DNA and the trimethylation of histone H3 lysine 27 (H3K27me3), which is critically involved in methylation and the regulation of affinity binding between histones and the DNA backbone [4–6]. Methylation of DNA is catalyzed by particular DNA methyltransferases (DNMTs), which use S-adenosyl-L-methionine (SAM) as the methyl group donor, which is involved in the repression of tumor suppressor genes including BRCA1, CDKN2A/p16, RASSF1A, GSTP1 [7, 8]. Similarly, methylation of histones on the lysine residue of H3 and H4 is catalyzed by histone methyltransferases (HMTs) through the methyl group donor SAM [9]. HMTs have a catalytic active SET domain that is highly specific to both histone residues and degrees of methylation. SAM serves as the endogenous co-substrate and binds to the SET domain of Enhancer of Zeste homolog 2 (EZH2), a polycomb group protein, to enable the transfer of a methyl group to the H3K27 substrate. Methylated K27 serves as a docking point for the recruitment of other polycomb group proteins, whose binding contributes to the formation of a repressive chromatin state with well-documented roles in regulating cellular function [10–12]. Aberrant EZH2 expression has been detected in several human malignancies and is involved in the inactivation of some target genes, including CDH1, DKK1, DAB2IP, and TIMP3, either dependent or independent of DNA methylation [13–15]. EZH2 physically interacts with and recruits DNMTs to methylate CpG and establishes a more deeply repressive chromatin state, resulting in transcriptional gene silencing [16]. The importance of this epigenetic alteration is demonstrated by the use of DNMT inhibitors *viz*. 5-azacytidine and 5-aza-2'-deoxycytidine (decitabine), which have well-characterized mechanisms of action against the treatment of hematological malignancies and solid tumors but have nonetheless been shown to be associated with serious side effects and toxicity [17, 18]. Pharmacologic inhibition of EZH2 is achieved by 3-deazaneplanocin-A (DZNep), a non-selective inhibitor of S-adenosylhomocysteine hydrolase, which disrupts methionine metabolism and inhibits cellular methyltransferase activity [19, 20]. Although the EZH2 catalytic inhibitor depletes H3K27me3 robustly, it does not seem to demonstrate potent efficacy in solid tumors. This highlights the importance of EZH2 and DNMTs as key epigenetic regulators and therapeutic targets that function through a common mechanistic pathway and silence gene expression related to the initiation and maintenance of cancer. Agents with a dual capability to effectively inhibit DNA methyltransferase and EZH2 enzyme activity might be potentially useful in the prevention and/or treatment of cancer. There is a continuing demand for new agents that are efficacious and minimally toxic. In recent years, epigenetic modification by plant-derived phytochemicals has been the focus of much cancer research.

Dietary flavonoids have received increasing attention as potential guardians against a variety of human diseases, including cancer [21–23]. Flavonoids are commonly divided into six flavone subclasses based on the connection position of the B and C rings and the degree of saturation, oxidation and hydroxylation of the C ring as flavonols, flavanones, flavon-3-ols (catechins), isoflavones and anthocynadins. In particular, the flavone subclass of flavonoids contains 15 carbon atoms present in a number of hydroxyl groups at the positions 3, 5, 7, 3', 4' and 5' [24]. Some naturally occurring flavones include Apigenin (4',5,7-trihydroxyflavone), Chrysin (5,7-dihydroxyflavone), Luteolin (3',4',5,7-tetrahydroxyflavone), Tangeritin (4',5,6,7,8-pentamethoxyflavone), Baicalein (5,6,7-trihydroxyflavone), Scutellarein (5,6,7,4'-tetrahydroxyflavone), and Wogonin (5,7-dihydroxy-8-methoxyflavone) [24]. Flavones are widely distributed in many herbs, fruits and vegetables and are substantial components of the human diet. They have been shown to possess a variety of biological characteristics [21–24]. Number of mechanisms have been attributed to plant flavones, including antioxidant properties and their influence on gene expression. The most common activity noted for majority of plant flavones include their role as potent antioxidants and free radical scavengers, with their biological activities related to anti-inflammatory, antimicrobial, antiviral, anti-mutagenic and anticancer functions. These biological activities are considered to be related to their interactions with several enzymes and proteins, including calcium phospholipid-dependent protein kinase, DNA topoisomerases, tyrosine protein kinase, phosphorylase kinase, phosphatidylinositol 3-kinase, cytochrome 1A1 expression and the total cellular glutathione level [25–27].

Recent studies have revealed that plant flavones interact with nucleic acids; these investigations on flavone-DNA interactions are of current interest and are thought to be important [28–30]. We previously demonstrated that the plant flavone Apigenin has affinity to accumulate in the nuclear matrix and binds to nucleic acid bases through mechanisms involving intercalation and/or minor groove binding [31]. These interactions between flavones and nucleic acid affect the activity of various proteins attached to DNA. Various plant flavonoids have been shown to inhibit the activity of DNMTs [32–34]; however, their dual effect on histones and DNA methylation has not been elucidated. To achieve the objective, we investigated the effect of plant flavones viz. Apigenin, Chrysin and Luteolin in inhibiting the methyltransferase activity of DNA and histone proteins. The compounds were subjected to UV-Vis spectroscopic analysis of interactions with calf-thymus DNA and sequence-dependent binding to duplex DNA in an aqueous solution at physiological conditions. Docking simulations were also performed employing the X-ray crystallographic structure of the DNMT1- and EZH2-flavone complex to determine the binding modes of these compounds. Through several biochemical assays, we additionally tested the ability of these plant flavones to inhibit DNA methylation in an artificial system using M.SssI as a substrate and histone H3 lysine 27 trimethylation on isolated nucleosomes.

## Materials and Methods

### Chemicals and Reagents

All chemicals and reagents were purchased from Sigma Chemical Co. (St. Louis, MO, USA) unless otherwise specified. Tissue culture supplies were procured from Falcon (Becton-Dickinson Labware, Franklin Lakes, NJ, USA). All tissue culture reagents were purchased from Invitrogen (Grand Island, NY, USA), whereas fetal bovine serum was purchased from Gemini Bio-Products (West Sacramento, CA, USA). Apigenin (>98% pure) was purchased from A.G. Scientific (San Diego, CA, USA), Luteolin (≥98% pure) was procured from Cayman Chemical (Ann Arbor, MI, USA), and Chrysin (>99% pure) was purchased from the Indofine Chemical Company (Hillsborough, NJ, USA).

### Cell Culture

Human prostate cancer LNCaP and DU145 cells and transformed human prostate epithelial RWPE-1 cells were obtained from the American Type Culture Collection (Manassas, VA, USA). LNCaP and DU145 cells were cultured in RPMI 1640 medium containing 10% fetal bovine serum supplemented with 1% penicillin-streptomycin at 37°C with 5% CO_2_. RWPE-1 cells were cultured in keratinocyte growth medium supplemented with 5 ng/ml human recombinant epidermal growth factor and 0.05 mg/ml bovine pituitary extract (Invitrogen, Carlsbad, CA, USA).

### Flavone Binding with DNA

Calf thymus (CT) DNA was prepared in double distilled water and adjusted to pH 7.2. It was then sonicated and filtered through a 0.45 μM filter. It stirred overnight at 4°C to obtain a homogeneous solution of polymerized DNA. An aqueous solution of flavones was freshly prepared. Experiments were performed in 0.1 M phosphate buffer solution, with pH 7.4. DNA solutions at 0.25 μM were prepared with flavones at a 0.5 mM concentration. The absorption spectra of all solutions were recorded from 230 nm to 500 nm using NanoDrop 1000.

### Flavone interactions with CG and AT-rich DNA Sequences

To analyze flavone interactions with DNA, 100 bp highly GC-rich oligos mimicking CpG islands ranging from -183 to-83 and AT-rich oligos from the -486 to-386 region of the GSTP1 promoter were synthesized. These single stranded oligonucleotides and their reverse complementary oligos were purchased from Invitrogen (Carlsbad, CA, USA). Stock solutions of each single strand were prepared at a 1 μg/μl concentration in Milli-Q water. Complementary oligonucleotides were mixed at equimolar concentrations and were annealed by bringing the solution to 95°C and allowing it cool slowly to room temperature. A 100 bp duplex DNA fragment was used as a DNA substrate (2.5 mM) for binding with 0.5 mM of Apigenin, Chrysin and Luteolin. Absorption spectrum changes were measured with a UV-Vis spectrophotometer in the 220–500 nm range in an aqueous solution at physiological conditions.

### Molecular Docking Studies

**Fig 1:**
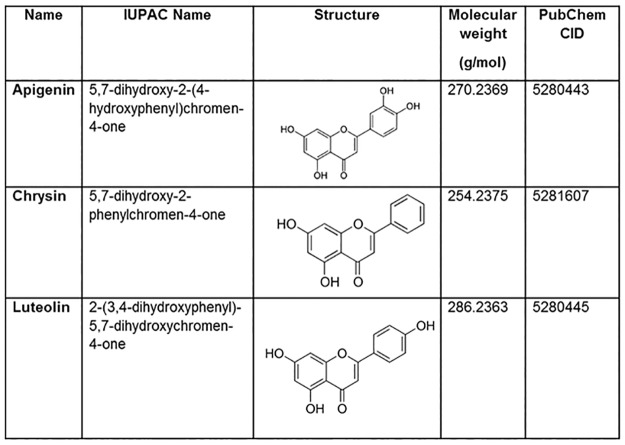
Ligands used for protein-ligand interaction analysis. The IUPAC name, structure, molecular weight and PubChem CID is provided for the ligands.

Three dimensional structural models for DNMT and EZH2 were built using a homology modeling technique. The protein sequence for human DNA (cytosine-5)-methyltransferase 1 (DNMT) was retrieved from UniProt (accession number P26358). Domain analysis for human DNMT using Pfam showed that the DNA methyltransferase catalytic domain spanned from residue number 1139 to 1597. The tertiary structure for this catalytic domain within DNMT was modelled using the Swiss-model server based on the three dimensional coordinates of DNMT1 (PDBID 3AV6) (doi: 10.1073/pnas.1019629108). For modeling EZH2, the BLAST program at NCBI was used to search for structures possessing sequences similar to EZH2. The crystal structure of the mixed lineage leukemia (mll1) set-2 domain was identified as the most similar sequence to EZH2 and was used as a template for modeling. The template structure was retrieved from a protein data bank (PDBID 2W5Y) (doi: 10.1016/j.molcel.2008.12.029). The Modeller program was used to build the structure of EZH2 based on satisfaction of homology and stereochemical restraints. The stereochemistry of the generated models for DNMT and EZH2 was validated using a Ramachandran plot. The structural models for DNMT and EZH2 were used for protein-ligand docking calculations using Schrodinger software (Schrodinger, LLC). A receptor energy grid was generated around the pockets of DNMT and EZH2 using Glide (Schrodinger, LLC). This energy grid defined the region for docking of ligands and was employed to evaluate the strength of protein-ligand interactions. Structures for three ligands (Fig 1), i.e., Apigenin (5,7,4'-trihydroxy-flavone), Luteolin (5,7,3',4'-tetrahydroxy-flavone), and Chrysin (5,7-dihydroxyflavone), were retrieved from the PubChem database and geometrically optimized using the LigPrep module (Schrodinger, LLC). These three compounds differed in the number of hydroxyl groups attached to the phenyl ring. All three ligands were individually docked to the DNMT and EZH2 binding pockets using Glide (Schrodinger, LLC) in an extra precision (XP) mode. The strength of binding between proteins and ligands was evaluated using GlideScore, which is an empirically derived scoring function, as previously described [35].

### DNA methyltransferase activity assay

Nuclear extracts from untreated, 5-Aza-dC, Apigenin-, Chrysin- and Luteolin-treated LNCaP cells were prepared using the EpiQuik Nuclear Extraction Kit (Epigentek, Farmingdale, NY, USA) per the manufacturer's protocol. The protein concentrations were estimated, and 20 μg nuclear lysate was used to measure DNMT activity or inhibition using the EpiQuik DNA Methyltransferase Activity Assay Kit (Epigentek) per the vendor's protocol.

### Histone methyltransferase activity assay

Total histone proteins from untreated DZNep, Apigenin, Chrysin and Luteolin were isolated from DU145 cells using an EpiQuik Total Histone Extraction Kit (Epigentek). Histone methyltransferase activities of H3K27me3 in treated and untreated samples were measured using a colorimetric quantification kit (Epigentek, Farmingdale, NY, USA) following the manufacturer’s protocol.

### *In vitro* promoter methylation and digestion with the methylation- sensitive HpaII enzyme

The substrate DNA for an *in vitro* methylation assay was a 721 bp fragment (-428/+243 relative to the initiation codon), which was amplified from RWPE-1 cells within the promoter region of the human GSTP1 gene. The methylation reactions were carried out in 1X M.SssI buffer with 160 mM SAM S-adenosylmethionine (supplied with M.SssI by New England Biolabs). For methylation, 1 μg of the purified GSTP1 promoter PCR product was incubated with 10 U of M.SssI methyltransferase enzyme (New England Biolabs, Ipswich, MA, USA) with or without flavones in 1X MSss1 buffer (50 mM NaCl, 10 mM Tris-HCl, 10 mM EDTA and 1 mM dithiothreitol), pH 8.0, for 3 h at 37°C in 50 μl of reaction volume. After completion, the reaction was inactivated at 65°C for 15 minutes and the DNA was purified using a QIAquick PCR Purification kit (Qiagen, Valencia, CA, USA). A total of 500 ng of purified DNA were digested for 3 h at 37°C with 20 units of HpaII (New England Biolabs) and was analyzed on 2% Tris-borate EDTA agarose gels.

### Bisulfite treatment and methyl-specific PCR

A 721 bp promoter fragment was isolated from RWPE-1 cells of the human GSTP1 gene as described above. The methylation reaction contained 1 μg substrate DNA and 10 units of M.SssI methylase (0.5 A mol/L, New England Biolabs, Ipswich, MA, USA) in a final volume of 50 μl. Flavones were added to final concentrations of 0.5 mmol/L, and the mixtures were then incubated at 37°C for 3 h. After completion, the reaction was inactivated at 65°C for 15 minutes and the DNA was purified using a QIAquick PCR Purification kit (Qiagen, Valencia, CA, USA). A total of 500 ng of *in vitro* methylated GSTP1 promoter DNA was used for bisulfite modification per the protocol provided with the EZ DNA Methylation–Gold Kit (Zymo, Orange County, CA, USA). This was followed by additional desalting and purification using the DNA Clean and Concentrator-5 Kit (Zymo). DNA was suspended in 10 μl of water and stored at -20°C. Primers to perform MS-PCR on the GSTP1 promoter were designed using Methyl Primer Express VR (Applied Biosystems, Foster City, CA, USA). A PCR reaction was performed using methylation-specific PCR (MSP) primer sequences that specifically recognized the methylated forward primer TTCGGGGTGTAGCGGTCGT and methylated reverse primer CCCCATACTAAAAACTCTAAACCCCATCCCC and the unmethylated forward primer GATGTTTGGGGTGTAGTGGTTGTT unmethylated reverse primer CCCCATACTAAAAACTCTAAACCCCATCCCC. PCR products were resolved in 2% agarose gels along with 1 Kb plus DNA ladder (Invitrogen, Carlsbad, CA, USA), and then were visualized and photographed with a Kodak Image Station 2000R.

### Histone isolation and Western blot analysis

Total histone proteins were isolated using an EpiQuik Total Histone Extraction Kit (Epigentek, Farmingdale, NY, USA). SDS/PAGE was performed on 4–20% acrylamide gels. The proteins were transferred to nitrocellulose membranes (Bio-Rad, Hercules, CA, USA) and probed with antibodies against histone H3K27me3 (Abcam Ab6147) and EZH2 (Abcam Ab5246S). Histone H3 (Cell Signaling, 9715) and β-actin (SantaCruz SC47778) were used as loading controls.

### Statistical Analysis

The experiments on cells, nuclear lysates and DNA were repeated at least three times. The results were expressed as the mean values ± SD. Statistical comparisons were made with an ANOVA followed by Dunnett’s multiple comparison test. *P* values <0.05 were considered significant.

## Results

**Fig 2:**
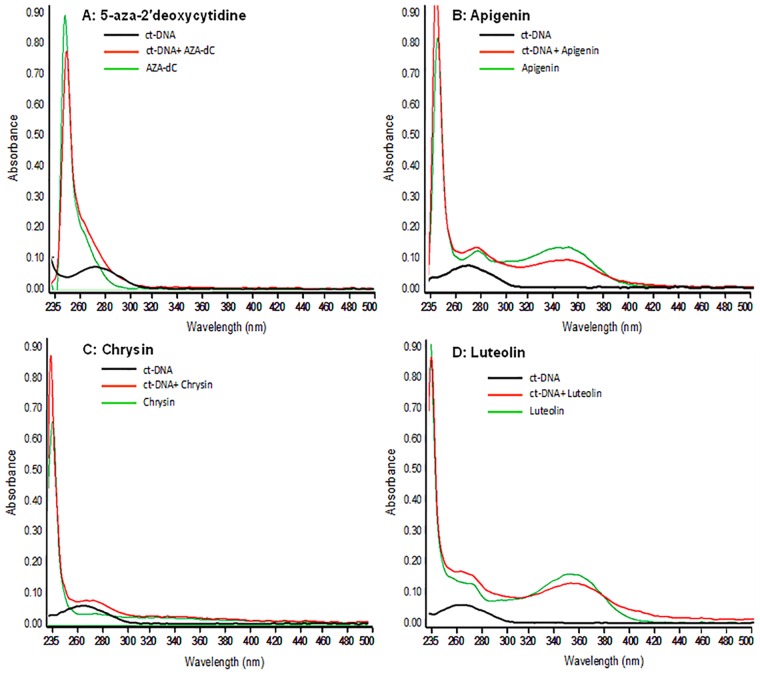
Interaction of dietary flavones with calf thymus (CT) DNA. (A) UV-Vis spectra of CT-DNA, with 5-aza-2’deoxycytidine, (B) Apigenin, (C) Chrysin, and (D) Luteolin. UV-Vis spectra of CT-DNA with flavones were performed in 0.1 M phosphate buffer solution, with pH 7.4. 0.25 μM DNA solutions were prepared with flavones at 0.5 mM concentration. Absorption spectra of the solutions were recorded from 230 nm to 500 nm using Nanodrop. The experiment was repeated three times with similar results. Details are described in the Materials and Methods section.

To study the interactions of plant flavones with DNA, calf thymus (ct)-DNA was used and the absorption spectra was recorded from 230 nm to 500 nm (Fig 2A–2D). Previous studies have demonstrated that intercalations of flavones into the DNA duplex cause major reductions in the intensity of the UV-Vis absorption band characteristics of flavones [36, 37]. Typically, two absorption bands are observed in the UV spectra of flavones: band I (300–420 nm), the absorption of the cinnamoyl part (B + C), and band II (250–285 nm), the conjugated system of ring A and ring C (γ-pyrone ring) in the molecule. Band I at higher wavelengths is related to the n−л* transitions whereas band II is related to the л−л* chromophoric transitions. As shown in Fig 2A–2D, changes in flavone spectra (0.5 mM) with added ct-DNA indicated the formation of some type of flavone-DNA complex. At pH 7.2, the UV-Vis spectra of Apigenin and Luteolin showed hyperchromic (264 nm) and hypochromic (354 nm) effects with the addition of ct-DNA. The absorbance of band II increases, *i*.*e*., the hyperchromic effect, whereas band I exhibits a decrease in absorbance (hypochromic effect). This hypochromic shift indicates helical ordering of flavones in the DNA helix. The UV-Vis band of Chrysin exhibited a hyperchromic effect at 274 nm, which was similar to that observed with 5-Aza-dC at 288 nm. These effects are indicative of flavone intercalation with the DNA duplex. The affinity of flavone-DNA binding seems to be driven by the number and positions of the–OH groups and is in the order Luteolin>Apigenin>Chrysin.

**Fig 3:**
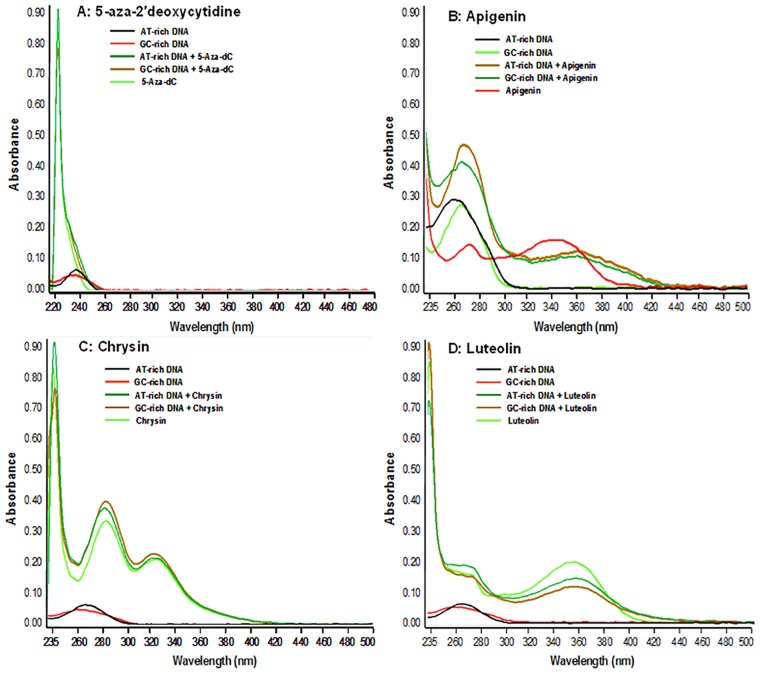
Interaction of dietary flavones with AT-rich and GC-rich DNA sequences. (A) UV-Vis spectra of AT-rich and GC-rich DNA sequences, with 5-aza-2’deoxycytidine, (B) Apigenin, (C) Chrysin, and (D) Luteolin. 100 bp highly GC-rich oligos mimicking CpG islands ranging from -183 to-83 and AT-rich oligos from the -486 to-386 region of the GSTP1 promoter were synthesized and used as a DNA substrate (2.5 mM) for binding with flavones at a 0.5 mM concentration. Absorption spectra of solutions were recorded from 220 nm to 500 nm using Nanodrop. The experiment was repeated three times with similar results. Details are described in the Materials and Methods section.

Next we determined the binding preference of plant flavones with AT- and GC-rich base pairs of nucleic acids. We synthesized 100 bp highly GC-rich sequence oligos and AT-rich oligos mimicking the GSTP1 gene. Fig 3A–3D shows the absorption spectra of plant flavones and 5-Aza-dC with AT-rich and GC-rich oligonucleotides. Two iso-absorptive points were observed in the absorption curves and ranged from 255–290 nm and 310–390 nm. The hyperchromic shift might be the reason for these oligos having an absorption at 270 nm. The hypochromic shift in the bands was in the order Luteolin> Apigenin> Chrysin, with preferential binding with the GC-rich sequence compared to AT-rich oligo nucleotides. No hypochromic shift in the bands was observed with 5-Aza-dC.

**Fig 4:**
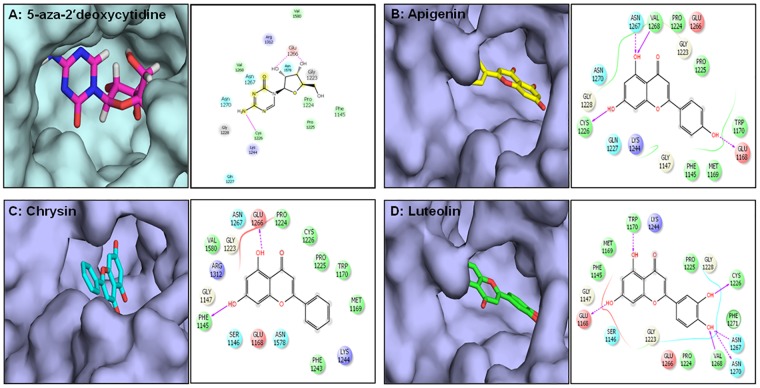
Molecular modeling of the interaction between dietary flavones and DNMT1. (A) 5-aza-2’deoxycytidine (in sticks) docked into the pocket of DNMT1 (shown as surface), (B) Apigenin, (C) Chrysin, and (D) Luteolin (Left panel). Schematic representation of different non-bonded interactions between ligands and amino acid residues of DNMT1 is shown in the right panel. The pink arrow represents a hydrogen bond and amino acids are colored according to their chemical characteristics. The Glide score for docking of 5-Aza-dC, Apigenin, Luteolin, and Chrysin was -7.16 Kcal/mol, -6.38 Kcal/mol, -5.85 Kcal/mol, and -7.34 Kcal/mol, respectively. Details are described in the Materials and Methods section.

Next, we performed *in silico* docking studies with plant flavones and 5-Aza-dC to determine their effectiveness in suppressing DNMT activity. Fig 4A shows a schematic representation of different non-bonded interactions between 5-Aza-dC and amino acid residues of DNMT1. 5-Aza-dC docking into the pocket of DNMT was stabilized by a combination of polar and non-polar interactions. The aromatic ring of the ligand formed a hydrogen bond with the side chain of Glu1266 through an amino group and a side chain of Asn1267 via a keto group. Additionally, a hydrogen bond was observed between one of the hydroxyl groups of ribose and the main chain of Cys1266. In addition, the ligand formed hydrophobic contacts with Pro1224, Pro1225, and Val1268. The Glide score for docking of 5-Aza-dC to DNMT1 was -7.16 Kcal/mol. Fig 4B shows the docked pose for Apigenin within the DNMT1 binding pocket, which was stabilized by three hydrogen bonds. The 4’ hydroxyl group participated in hydrogen bonding with a side chain carboxylic group of Glu1168. On the other end of the ligand, the hydroxyl group attached to C5 formed a hydrogen bond with a side chain of Asn1267 and backbone carbonyl group of Val1268. In addition, the hydroxyl group attached to C7 participated in hydrogen bonding with the backbone of Cys1226. The GlideScore for docking of Apigenin to DNMT1 was -6.38 Kcal/mol. Chrysin has two hydroxyl groups, and the docked pose showed that both of these groups were engaged in intermolecular hydrogen bonding (Fig 4C). The C5 hydroxyl groups acted as hydrogen bond donors to interact with the carboxylic side chain of Glu1266. The hydroxyl group attached to C7 formed a hydrogen bond with the backbone carboxylic group of Phe1145. The GlideScore for Chrysin docking to DNMT was -5.85 Kcal/mol. The binding of Luteolin was stabilized by a total of six hydrogen bond interactions with the DNMT residues lining the binding pocket (Fig 4D). All four hydroxyl groups were engaged in inter-molecular hydrogen bonding with different amino acids. The hydroxyl groups attached to C5 and C7 formed hydrogen bonds with side chains of Trp1170 and Glu1168, respectively. The hydroxyl group attached to C3’ participated in hydrogen bonding with the backbone of Cys1226. The C4’ hydroxyl group was observed to be within hydrogen bonding distance from three residues: Asn1267, Val1268, and Asn1270. Overall, because of the number of favorable intermolecular interactions, the GlideScore for docking of Luteolin to DNMT was -7.34 Kcal/mol. Overall, *in silico* protein-ligand docking simulations showed that all three flavones interact with DNMT with comparable binding strengths. The relative strength of binding was observed to be Luteolin>Apigenin>Chrysin.

**Fig 5:**
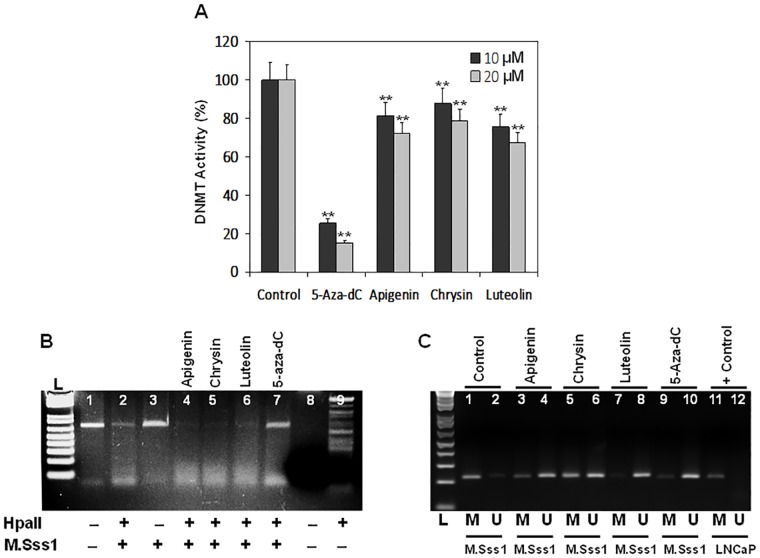
Effect of dietary flavones on DNA methyltransferase activity and *in vitro* methylation. (A) Dose-dependent inhibition of DNMT activity by 10- μM and 20-μM concentrations of 5-Aza-dC, Apigenin, Chrysin and Luteolin. The results are the mean of 3–4 determinations and were analyzed with a one way ANOVA, bars ± SD. **P<0.001 (B) Inhibition of purified recombinant DNA methyltransferase activity by dietary flavones. A 728 bp fragment (-428/+243 relative to the initiation codon) isolated from RWPE-1 cells within the promoter region of the human GSTP1 gene was used as substrate DNA. The methylation reactions were carried out in 1X M.SssI buffer with 160 mM SAM S-adinosylmethionine. The addition of 20 μg of flavones resulted in a detectable decrease in DNA methyltransferase activity (Lanes 4–6), compared to controls (Lane 2). No inhibitory affect was observed with an equal concentration of 5-Aza-dC. (C) MS-PCR for GSTP1 promoter on genomic DNA isolated from RWPE1 cells after treatment with 20 μM of 5-Aza-dC and dietary flavones for 48 h and treatment with methylation using M.SssI. The products generated with primers specific for unmethylated GSTP1 CpG island alleles (U) and for hypermethylated GSTP1 CpG island alleles (M) are displayed. L, DNA ladder. Details are described in the Materials and Methods section.

In the next set of experiments, we determined the potential of plant flavones in inhibiting DNA methylation and DNMT enzyme activity. First, we performed a DNMT inhibition assay with nuclear extracts after treatment of human prostate cancer LNCaP cells with 10 μM and 20 μM of plant flavones for 48 h. A dose-dependent inhibition of DNMT activity was observed with each flavone in the order Luteolin>Apigenin>Chrysin. 5-Aza-dC was used as a positive control (Fig 5A). In the next series of experiments, we analyzed the effect of flavones on inhibiting DNA methylation *in vitro* (Fig 5B). For this assay, we used purified recombinant CpG methylase M.SssI. This enzyme is distinguished by robust activity and shows significant structural similarities with the DNMT1 catalytic domain. A 721 bp PCR fragment from the promoter region of the human GSTP1 gene was used as a substrate, and DNA methylation was visualized by digestion with the methylation-sensitive restriction enzyme HpaII (Lane 2). The inhibition of DNMTs can be detected by the appearance of unprotected smaller restriction fragments upon exposure with flavones, as shown in lanes 4–6. A direct comparison of plant flavones and 5-Aza-dC in this assay revealed a readily detectable inhibitory effect for flavones, but not for 5-Aza-dC (Lane 7). In the last experiment, we determined the demethylation potential of plant flavones. For this study, the 1.5 kb promoter present within the second CpG islands of the GSTP1 gene was applied, which is hypermethylated in prostate cancer cells. We evaluated the effect of plant flavones in demethylating the particular gene fragment by utilizing artificial methylation with M.Sss1. As shown in fig 5C, methyl-specific PCR on the proximal GSTP1 promoter revealed that flavones have the ability to demethylate the promoter sequence by increasing the unmethylated fraction, which was more pronounced with Luteolin followed by Apigenin and Chrysin, respectively.

**Fig 6:**
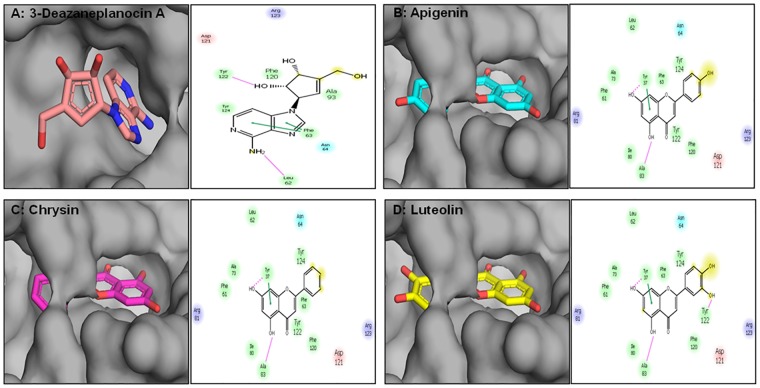
Molecular modeling of the interaction between dietary flavones and EZH2. (A) 3-Deazaneplanocin A (in sticks) docked into the active pocket of EZH2 (shown as surface), (B) Apigenin, (C) Chrysin, and (D) Luteolin (Left panel). Schematic representation of different non-bonded interactions between ligand and amino acid residues of EZH2 is shown in the right panel. The pink arrow represents a hydrogen bond and amino acids are colored according to their chemical characteristics. The Glide scores for docking of DZNep, Apigenin, Luteolin, and Chrysin were -7.62 Kcal/mol, -10.07 Kcal/mol, -9.73 Kcal/mol, and -11.23 Kcal/mol, respectively. Details are described in the Materials and Methods section.

The histone methyltransferase catalyzes methyl group transfer from a universal methyl donor, SAM, to the nitrogen atom of the lysine side chain, forming *S*-adenosyl-_L_-homocysteine (SAH) as a product [14, 15]. The catalytic domain is conserved among the methyltransferases, which consists of approximately 130 amino acids, designated as the SET domain. EZH2 contains the conserved SET domain with catalytic function [16]. To determine whether flavones have the ability to inhibit EZH2 methyltrasferase activity, we performed docking simulation studies using the crystal structure of EZH2. First, we conducted studies docking DZNep to the catalytic pocket of EZH2 (Fig 6A). DZNep docking to EZH2 was stabilized by a diverse set of non-bonded interactions, including hydrogen bonds between the amino acid and one of the hydroxyl groups in the ligand with the backbone of Leu62 and Tyr122, respectively. In addition, DZNep formed contacts with six non-polar amino acids and three polar amino acids (Fig 6A, Right panel). The Glide score for the docking of DZNep to EZH2 was -7.62 Kcal/mol. Apigenin binding to the EZH2 was stabilized primarily by non-polar interactions (Fig 6B). Ten different residues of EZH2 participated in non-bonded interactions with Apigenin and two hydroxyl groups on the same ring in the ligand formed a hydrogen bond with Tyr37 and Ala83, respectively. Additionally, some polar amino acids, such as Arg81, Asp121, and Arg123, showed favorable interactions with the ligand. The Glide score for the docking of Apigenin to EZH2 was -10.07 Kcal/mol. Chrysin docking to EZH2 was very similar to that of Apigenin with a Glide score of -9.73 Kcal/mol (Fig 6C). The right panel in Fig 6C shows the docked structure of Chrysin to EZH2 and its non-bonded interactions with the protein. Luteolin has an additional hydroxyl group in its chemical structure compared to Apigenin. The docking results showed that the presence of an additional hydroxyl group enhanced the interaction between the ligand and protein. Apart from the non-bonded interactions observed in the case of Apigenin, Luteolin formed an additional hydrogen bond with the backbone of Tyr122 (Fig 6D). The Glide score for the docking of Luteolin to EZH2 was -11.23 Kcal/mol.

**Fig 7:**
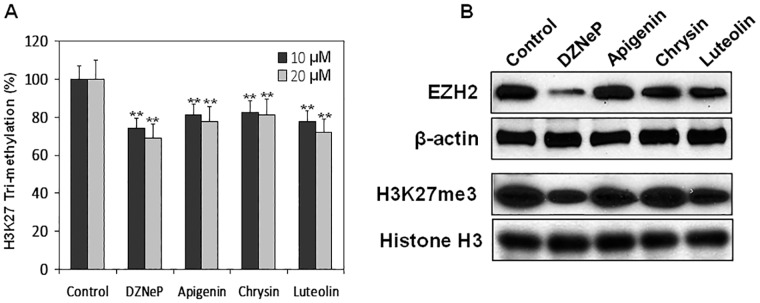
Effect of dietary flavones on EZH2 catalytic activity and EZH2 and H3K27me3 protein expression. (A) Dose-dependent inhibition of EZH2 catalytic activity measured through H3K27me3 with 10- μM and 20-μM concentrations of DZNep, Apigenin, Chrysin and Luteolin. The results are the mean of 3–4 determinations and were analyzed with a one way ANOVA, bars ± SD. **P<0.001. (B) Inhibition of EZH2 and H3K27me3 protein expression after treatment with 20 μM DZNep, Apigenin, Chrysin and Luteolin for 48 h. Anti-β-actin and anti-histone H3 were used as loading controls.

Next, we determined the potential of flavones in inhibiting H3K27me3 activity and the protein expression of EZH2. We performed H3K27me3 inhibition assay with histone extracts after treatment of human prostate cancer DU145 cells with 10 and 20 μM of individual plant flavones. A dose-dependent inhibition of H3K27me3 activity was observed with each flavone in the order Luteolin>Apigenin>Chrysin, where DZNep was used as a positive control (Fig 7A). We also performed western blotting to determine the effect of flavones on EZH2 protein expression and H3K27me3 using histone protein isolated from DU145 cells treated with flavones. As shown in Fig 7B, treatment of DU145 cells with 20 μM flavones for 48 h resulted in a marked decrease in EZH2 protein expression compared to the controls. Similar results were noted with H3K27me3, where flavones demonstrated their ability to inhibit H3K27me3 in histone proteins.

## Discussion

In this study, we explored the dual interactions of flavones in inhibiting the methylation of genomic DNA and the trimethylation of lysine 27 at histone H3 using various artificial techniques. Our present study clearly demonstrated that flavones have the ability to reverse both DNA methylation and the trimethylation of lysine 27 at the H3 histone both in cultured cells and in an artificial *in vitro* system.

The essential role of epigenetic defects in carcinogenesis has been established over the last decade [4–6]. Aberrant methylation is a mechanism by which tumor suppressor genes are inactivated; it is commonly observed in tumor tissues and cancer cell lines and primarily achieved through DNA methylation [38, 39]. DNA methylation at the C5 position of cytosine affects gene expression in many biological processes driven by DNA methyltrasferases, including DNMT1, DNMT2, DNMT3a and DNMT3b [4–6]. The catalytic domains of the four human DNMT enzymes are highly conserved and they interact similarly with DNMT1. Pharmacologic inhibitors of DNMT that are currently in use in the clinic are non-selective cytosine analogs with significant cytotoxic effects. A general consensus is that 5-Aza-dC remains the most potent DNMT inhibitor [40]. Several natural agents belonging to diverse chemical classes have indicated DNA binding activity [28–30]. Molecular modeling studies suggested that hydroxyl groups in the B ring of Apigenin and Luteolin bind efficiently to the catalytic pocket of DNMT1 and have a strong dependency on binding energy in the presence of the hydroxyl group. In addition, flavones offer some advantages over 5-Aza-dC as a novel non-nucleoside inhibitor of DNMT1 because they are known to rapidly intercalate with DNA [36, 37]. In our study, DNMT inhibition by plant flavones seems to be due to the binding of flavones at the catalytic binding pocket of DNMTs. These might be different from catechol-containing polyphenols, such as epigallocatechic-3-gallate, in which the noncompetitive inhibition of DNA methylation catalyzed by DNMTs is majorly due to the higher levels of SAM resulting from the catechol-O-methyltransferase-mediated O-methylation of these compounds [41]. Further studies showed inhibition in DNMT activity in the nuclear extracts from LNCaP cells and in artificial systems of methylation initiated by M.SssI, which was similar to that observed by 5-Aza-dC. The precise molecular mechanisms remain to be analyzed in further experiments.

Over the last decade, much attention has been focused on the binding of small molecules to DNA, as a result of the advantage of these molecules as potential drugs [28–30, 36, 37]. Many natural agents have served as analogues in research on protein-nucleic acid recognition, and they provide site-specific reagents for molecular biology. Therefore, the investigation of drug-DNA interactions is important for identifying the mechanisms of the drug action and designing specific targeting drugs. In this regard, the flavonoid scaffold plays an important role in the interactions of these molecules with DNA, particularly through the presence of a planar chromophore, which is possibly capable of intercalation between base pairs. Our results showed that the intercalation of flavones with calf thymus DNA causes major reductions in the intensity of the UV-Vis absorption band characteristics between 260–280 nm, where Apigenin and Luteolin with the 1 and 2 hydroxy groups in the B ring resulted in significant intercalation with DNA. Chrysin (no hydroxyl group in the B ring) resulted in less significant DNA binding. This further strengthens the argument that phenolics, hydroxyl groups on the B ring, are essential for the binding of flavones to nucleic acids. Apigenin and Luteolin exhibited a higher binding constant than Chrysin, which suggested that the carbonyl group of DNA bases was involved in fairly strong hydrogen bonding with these flavones. Because Apigenin and Luteolin are less water soluble than Chrysin, they are less solvated and consequently susceptible to intercalation within DNA. Experimental data from previous studies indicated that Luteolin-DNA and Apigenin-DNA interactions might be complex and may involve external groove binding in addition to intercalation [28, 37, 42]. Evidence for grove binding comes from infrared spectroscopy, where the mini groove binding of flavones occurs on the DNA [37, 42]. Flavones form a DNA complex that can adjust well to the groove of the DNA, with a binding site of three base pairs and preferentially involving GC residues [43]. Further studies are required to optimize major groove recognition by flavones under physiological conditions.

Multiple structure affinity relationship studies have further demonstrated that flavones are capable of intercalating into DNA duplexes [44, 45]. The planarity of the benzopyrone skeleton of flavones allows them to easily intercalate the DNA helixes compared to other less planar and non-polar flavonoids. In addition, flavones have been found to promote higher-order DNA transitions (B to A-DNA) upon complexation, thus suggesting the possibility of preferential binding to sequences that are associated with these confirmations. To determine which sites of flavones play a role in DNA complexation and preferential binding with nucleic acids, we examined the UV-Vis spectra of artificially synthesized 100 bp GC- and AT-rich sequences mimicking the human GSTP1 gene. In our studies, the observed decrease in UV-Vis intensities may be associated with the preferential binding of flavones such as Apigenin and Luteolin to GC-rich sequences instead of to AT-rich DNA. This is an important observation because it has been shown that cytosine followed by guanines are primary sites for the addition of a proton (H+) in DNA. This process of protonation causes more changes in GC-rich DNA than AT-rich DNA. Natural DNAs comprising both GC and AT base pairs undergo protonation-induced structural changes, regardless of their base composition. In such cases, hydrogen bonding and electrostatic binding occur with the phosphate strands and the GC-rich base pair, as performed through docking simulation studies. Thus, these electrostatic interactions are important for achieving high affinity with GC-rich bases. Further in-depth studies are required on sequences having different GC and AT contents to investigate possible sequence selectivity by plant flavones.

**Fig 8:**
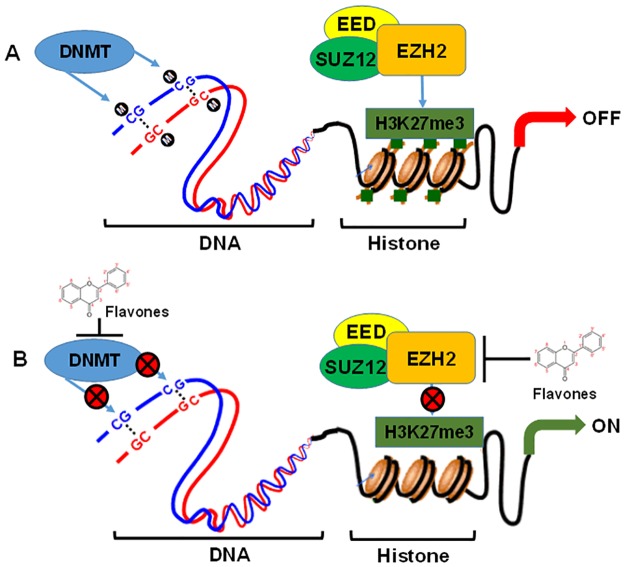
Proposed model of the dual action of dietary flavones in inhibiting DNA methyltransferase and histone methyltransferase. (A) The two major epigenetic mechanisms, DNA methylation and histone methylation, acted in concert to regulate gene transcription. The DNA double helix backbone is shown in blue and red. In DNA methylation, methyl groups are added to a cytosine that is immediately 5′ to a guanine. In general, an increase in DNA methylation leads to a decrease in gene transcription. Histone methylation is accomplished by trimethylation of histone H3 lysine 27 (H3K27me3), which is catalyzed by the enhancer of zeste homolog 2 enzyme (EZH2) resulting in gene transcriptional repression; occurring to the tails of histones (histones shown in brown, with green H3K27 marks on tails), leading to a condensed chromatin state. (B) Dietary flavones viz. Apigenin, Chrysin and Luteolin can bind to the DNA bases, dock on the catalytic pocket of DNMT and HMT to inhibit methylation resulting in relaxed chromatin, increasing the likelihood of gene transcription.

The genes that are silenced during cancer progression comprise tumor suppressor genes, regulatory genes and genes involved in differentiation [38, 45]. These genes are often inactivated by epigenetic mechanisms involving the methylation of cytosine in CpG islands of the promoter DNA and modification of the histone proteins. Common histone modifications leading to gene silencing in cancer include histone deacetylation, histone H3 lysine 9 methylation, and histone H3 lysine 27 trimethylation (H3K27me3). EZH2 is a catalytic subunit of the polycomb repressive complex 2 (PRC2) that represses gene expression by H3K27me3 [8–12]. Because of the EZH2-mediated effect in gene silencing and its over-expression in a broad spectrum of human tumors, it is an important drug discovery target. To date, no pharmacologic agent inhibiting EZH2 has been approved for its use in the clinic, and considerable effort has been exerted in developing EZH2 methyltransferase inhibitors. Although EZH2-mediated methylation also contributes as a potential independent mechanism for epigenetic gene silencing, it also cooperates with other enzymes that participate in epigenetic gene silencing. A physical and functional relationship between EZH2 and DNMTs has been recently documented, suggesting the potential interaction between these different classes of epigenetic modifying enzymes controlling gene expression [16, 46]. We initiated studies on plant flavones due to their ability to block the active site of the human DNMT1 and EZH2 in an in silico model. Other obvious reasons for selection of plant flavones are the double bond between C2 and C3 of the flavone skeleton, which allows extended conjugation among the three rings and forms an integrated π-conjugated plane that facilitates its binding to DNA. The presence of the 3'-OH group was shown to cause a marked increase in the fraction of bound DNA, as with Apigenin and Luteolin. Our in silico studies, we further demonstrated the ability of flavones to bind to the catalytic pocket of EZH2 and, in a cell culture study with nucleotides obtained from cancer cells, flavones demonstrated the ability to inhibit EZH2 enzyme activity and protein expression. Together, our study demonstrated the dual potential of flavones in targeting DNMTs and EZH2, which is better than targeting a single silencing enzyme. The molecular representation of this effect by plant flavones is shown in Fig 8. The combination of inhibitors simultaneously targeting DNA methylation and histone methylation is particularly promising in light of recent findings that exhibit synergistic loss of clonogenicity in human leukemia HL-60 cells and remarkable activation of tumor suppressor genes [47]. In another study, a combination of 5-Aza-dC and GSK126 had similar comparable effects on the growth of human breast cancer cells both in culture and in an *in vivo* model [48]. In this study, combined treatment with these two agents induced the marked re-activation of some tumor suppressor genes exhibiting an additive inhibitory effect on tumor growth. These studies prompted the quest to identify agents that are minimally toxic and are dual inhibitors of DNA and histone methylation. Additional studies are required to determine the dual modification in gene expression after treating tumor cells with various flavones.

Flavones are a class of naturally occurring phenolic plant compounds that exhibit biological and pharmacological activity coupled with low toxicity. These compounds are widely distributed in the plant kingdom and are ingested daily by humans. Numerous case-controlled and cohort studies have evaluated the association between flavonoid intake and the risk of several human cancers, including breast [49], colon [50], prostate [51], lung [52], liver [53] and ovary [54]. These studies provided inconsistent results as evidence for an association of flavonoids with decreased cancer risk. However, the association with Apigenin intake was statistically significant in the model adjusted for multiple covariates. In another study, the inverse association with the intake of the plant flavones Kaempferol and Luteolin in reducing ovarian cancer risk was strongest compared to other flavonoids [54]. Similar results were obtained in two separate studies conducted in Greece, where a strong statistically significant adverse association of flavone intake was observed in breast and hepatocellular carcinomas [49, 53].

Recently, data have been presented on the interaction of flavones with various epigenetic silencing enzymes [55]. Based on our spectroscopic study of the interaction of flavones with calf-thymus DNA and their preferential binding with GC-rich and AT-rich sequences, we suggest classic intercalation is the dominant binding mode and may affect reactions associated with enzymes on DNA molecules. Furthermore, *in silico* docking studies with flavones on DNMT1 and EZH2 catalytic sites and several biochemical assays demonstrated the ability of flavones to inhibit DNA methylation and histone H3K27me3 in an artificial system, which further attested to their anticancer properties at the epigenetic level. Although several mechanisms by which flavones might prevent cancer have been confirmed or are under investigation, our data are consistent with the concept that their activity on epigenetic silencing enzymes accounts for their documented capacity to serve as preventive/therapeutic agents against various human cancers, at least in part, by interfering with epigenetic pathways.

## Abbreviations

DNMTs: DNA methyltransferases
HMTs: histone methyltransferases
EZH2: Enhancer of zeste homolog 2
H3K27me3: trimethylation of histone H3 lysine 27
SAM: S-adenosyl-L-methionine
SAH: S-adenosyl-L-homocysteine
RASSF1A: Ras association domain-containing protein 1
GSTP1: Glutathione S-Transferase Pi 1
DKK1: Dickkopf WNT Signaling Pathway Inhibitor 1
DAB2IP: DOC-2/DAB2 interactive protein
TIMP3: tissue inhibitor of metalloproteinases 3
5-Aza-dC: 5-aza-2’-deoxycytidine
DZNep: 3-deazaneplanocin-A
