# Correction: Dietary Flavones as Dual Inhibitors of DNA Methyltransferases and Histone Methyltransferases

**DOI:** 10.1371/journal.pone.0167897

**Published:** 2016-12-01

**Authors:** Rajnee Kanwal, Manish Datt, Xiaoqi Liu, Sanjay Gupta

In Fig 1, the structures of Apigenin and Luteolin are incorrectly swapped. Please see the correct Fig 1 and its caption below.

**Fig 1:**
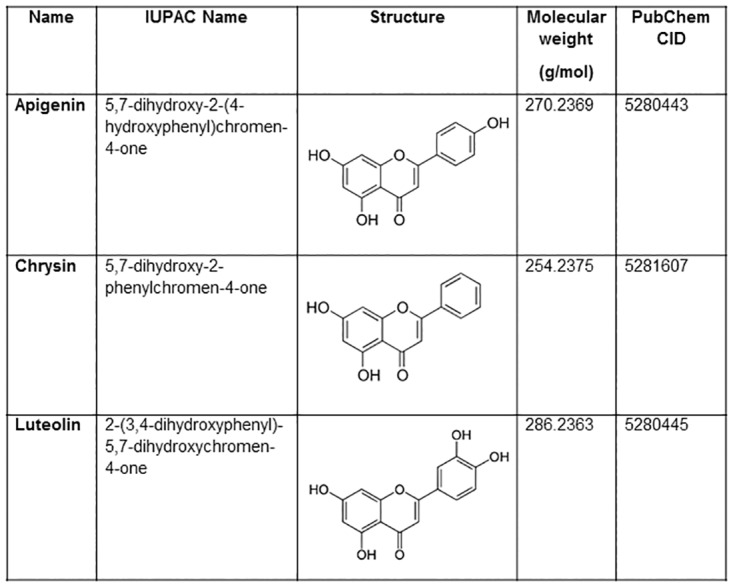
Ligands used for protein-ligand interaction analysis. The IUPAC name, structure, molecular weight and PubChem CID is provided for the ligands.
